# (UN)EQUAL BURDEN OF CARE: STAFF PERSPECTIVES ON NURSING HOME CARE AND QUALITY OF LIFE FOR RESIDENTS OF COLOR

**DOI:** 10.1093/geroni/igz038.2872

**Published:** 2019-11-08

**Authors:** Odichinma C Akosionu, Tetyana P Shippee, Heather Davila, Mai See Thao, Moses Waiswa, Tricia Skarphol

**Affiliations:** 1 University of Minnesota, Minneapolis, Minnesota, United States; 2 VA Boston Healthcare System, Boston, Massachusetts, United States; 3 Medical College of Wisconsin, Wauwatosa, Wisconsin, United States

## Abstract

Racial disparities in quality of care (QoC) and quality of life (QoL) for nursing home (NH) residents persist even as the proportion of minorities is significantly increasing. Staff of color are a growing part of the long-term care workforce and staffing is a key component for delivering quality care. This study looks at staff (n=60) perspectives on resident QOL through semi-structured interviews, using thematic analysis in six Minnesota high proportion minority NHs. Key findings show that staff of color are concerned about the QoC and QoL residents of color experience, and take extra steps to provide care that goes beyond addressing their clinical needs. This agency of providing extra care is a factor in burnout among staff of color. More research on how this unequal burden of care impacts QoC/QoL is important to address the disproportionate role that staff of color play in reducing disparities in resident QoC and QoL.

